# The capacity of primary health care facilities in Saudi Arabia: infrastructure, services, drug availability, and human resources

**DOI:** 10.1186/s12913-021-06355-x

**Published:** 2021-04-20

**Authors:** Quds Al Saffer, Taghred Al-Ghaith, Ahlam Alshehri, Rimah Al-Mohammed, Shahad Al Homidi, Mariam M. Hamza, Christopher H. Herbst, Nahar Alazemi

**Affiliations:** 1 Saudi Health Council, Riyadh, Saudi Arabia; 2 World Bank Group, Washington, DC USA

**Keywords:** Saudi Arabia, Primary care, Human resources for health, Readiness, Service availability, Gulf

## Abstract

**Background:**

Primary healthcare (PHC) is an essential component of an effective healthcare system. The Kingdom of Saudi Arabia’s (KSA) health reforms prioritize tackling the increasing noncommunicable disease burden by prioritizing PHC, centering it as the core of the newly proposed *Model of Care.* To identify challenges and opportunities to scale up PHC capacity, understanding the current capacity of primary health care centers (PHCC) is critical. A limited number of publications review PHC capacity in KSA, focusing on specific regions/sectors; this paper is a first to examine PHC capacity on a national level.

**Methods:**

The study uses a countrywide Facility Survey that collected data in 2018 from 2319 PHCCs, generating information on their characteristics, number of health workers, services provided, and capacity elements captured through the Service Availability and Drug Availability constructed indices. Descriptive analysis was performed by rural-urban classification. Ordinary Least Squares (OLS) regressions were used to understand correlates to health workers and equipment availability. Finally, a logistic regression was fitted for selected services. Regressions controlled for various measures to determine correlates with facilities’ capacity.

**Results:**

On a national level, there are 0.74 PHCCs per 10,000 population in KSA. There are variations in the distribution of PHCCs across regions and within regions across rural and urban areas. PHCCs in urban areas have more examination rooms but lower examination room densities. Offering 24 × 7 services in PHCCs is infrequent and dependency on paper-based medical recording remains common. More urban regions are more likely to offer general services but less likely to offer burn management and emergency services. PHCCs are mostly staffed with general medicine, family medicine, and obstetrics & gynecology physicians, whose numbers are more concentrated in urban areas; however, their densities are higher in rural areas. Finally, psychiatrists and nutritionists are rare to find in PHCCs.

**Conclusions:**

Decision-makers need to consider several factors when designing PHC policies. For instance, PHC accreditation needs to be prioritized given its positive correlation with service provision and health workers availability. PHC 24 × 7 operation also needs considerations in rural areas due to the high dependency on PHCCs. Finally, there is a substantial need for improvements in e-health.

## Background

Primary healthcare centers (PHCCs) are considered the first line of interaction between the patient and the healthcare system. Many countries include health indicators concerning primary healthcare quality or operation to measure their healthcare system [1]. From the Alma-Ata declaration of 1978, on which most of the principles underpinning primary healthcare rests, to the Astana Declaration of 2018, unanimously endorsed by all WHO Member states, significant emphasis is placed on the importance of primary healthcare. In both declarations, primary healthcare is an essential component of promoting health and health outcomes and is seen as a foundation of an effective and responsive healthcare system [2]. Countries that have adopted a primary healthcare focus have made remarkable progress toward the Sustainable Development Goals (SDGs) agenda, whether they are resource-rich or resource-poor countries [1–3].

For these reasons, investment in primary healthcare is a priority, especially with the evidence showing its vital role in improving the population’s health and lowering health expenditure. Nonetheless, even in countries known for having excellent healthcare systems like the UK [4], still faces difficulty with PHCs accessibility and services provided among its population, particularly for the rural population [5]. The UK is not the only case; many other countries like India, China, and Australia are also sustaining the same hardships [6, 7]. This limitation in rural areas across countries is multidimensional problem that include availability and distribution of the centers, their infrastructure, health workers, services provided and medications [8, 9].

The Kingdom of Saudi Arabia’s (KSA) health sector, like those of many other countries globally, is experiencing critical challenges because of (1) increased demand for health services given its growing population, (2) the high cost of healthcare services, (3) inequitable access, and (4) concerns regarding the quality and safety of care [4]. In KSA, the sector is thus undergoing rapid reform in line with the country’s National Transformation Program, as part of Vision 2030—Saudi Arabia’s vision for the future—by adopting three pillars that will lay the foundation for successfully achieving this vision: (1) facilitate access to health services, (2) improve the quality and efficiency of health services, and (3) promote health risk prevention. Saudi Arabia has already started shifting focus and investment from secondary and tertiary healthcare facilities toward reforming and restructuring primary healthcare, aiming to realize these goals [10].

Saudi Arabia, similar to other developing and developed countries, is exhibiting a similar shift when it comes to health status and disease burden from communicable. National statistics estimated that the common disease which leading to death in Kingdome is non-communicable diseases. Cardiovascular diseases account for the most deaths in is the Kingdom; diabetes also comes high on the list, which can be attributed to the significantly high obesity levels. in Saudi Arabia. Furthermore, traffic-related, transportation deaths and injuries are very high, which puts tremendous pressure on the health system [11]. The current disease burden, which is becoming increasingly comprised of noncommunicable diseases [12], is yet another reason that KSA should be prioritizing healthcare spending toward strengthening its primary and preventive care to achieve efficiency and value for money. The proposed reforms, with the new model of care, envision this priority, which is why it is important to understand the national readiness of PHCCs to implement such reforms.

Saudi Arabia has been working towards incorporating preventive and primary curative healthcare services through PHCs, covering extensive services. These services include controlling infectious diseases through immunization, child and maternity health, basic dental services, chronic disease management and follow-up, essential medications in addition to basic dental services, and health education [13].

The published research concerning primary healthcare in Saudi Arabia is considered scarce [14]. Most of the published studies on this subject in Saudi Arabia focus on chronic and infectious diseases [14–18], physician knowledge and perception of specific care modules [15, 16, 19], and—to measure quality of care— patient satisfaction of primary healthcare services and awareness of certain topics such as contraception [17, 19–21]. Very few articles discuss the capacity of PHCCs and all are limited to a specific healthcare sector and/or limited regions in Saudi Arabia [14, 22].

For policymakers or stakeholders to make informed decisions, a detailed assessment of the health system must be available. A current gap on the national situation of primary care exists. The objective of this study is to fill this gap, assessing the distribution of PHCCs across the Kingdom and their infrastructures, available workforce, medication, and type of services provided to aid in determining the pathway and focus of future policies and initiatives for enhancing primary healthcare in Saudi Arabia. This study is the first national study that includes data collected from all healthcare sectors in Saudi Arabia and all 13 administrative regions.

The Facility Survey used in this study was a country-level survey undertaken as part of the Balanced Distribution of Healthcare Facilities in Saudi Arabia project, at the Saudi Health council, [23] to gather and analyze data from all KSA facilities in order to assess the service availability and readiness of health facilities to manage the increasing disease burden and patient load. The key objective of the survey was to support stakeholders and policymakers in evidence-based decision-making and in developing better-informed annual operational plans. In broader terms, the survey’s goal was to support national planners in planning and managing health systems—for example, in assessing the equitable and appropriate distribution of services, human resources, and availability of medicines and supplies. The survey was part of a comprehensive study, which included interviews with stakeholders.

For any policymaker or a stakeholder to make informed decisions, primary healthcare’s current situation must be examined. The distribution of PHC across the kingdom and their infrastructures and only one factor assessing the capacity. A closer look at the available workforce, medication, and type of services provided are essential in determining the pathway and focus of future policies and initiatives for enhancing primary healthcare in Saudi Arabia.

## Methods

### Data and sampling methods

The study relies on The Saudi Balanced Distribution data collected by the Saudi Health Council (SHC). This country-level facility survey, part of a broader study was designed to assess the primary healthcare facilities’ capacity in KSA in the following aspects: infrastructure, services, drug availability, and human resources. The data requested were 2017 data and the collection occurred between June 2018 and April 2019 [23].

The first step in developing the health survey was to develop a master facility list: a catalogue of administrative information and information that identifies each facility (signature domain), as well as basic information on the service capacity of each facility (service domain). To develop the master list, official letters from the SHC were sent to all governmental and private healthcare entities to obtain a catalogue of all the facilities under them. After receiving the catalogues from all healthcare sectors, a draft of the master list was developed; duplicates were removed; and each facility was classified according to its sector, type, and geographic location. The final master list contained 6274 facilities, of which 2398 were PHCCs.

The Facility Survey was designed to assess infrastructure, services, and human resources for all healthcare facilities considering the special characteristic and type of services each facility type provides. The survey had three main sections: infrastructure, services, and human resources. The services and infrastructure sections adopted some elements from the Service Availability and Readiness Assessment (SARA), with some modifications to tailor it to the Saudi context [24]. For health human resources classifications, the local Saudi Commission for Health Specialties (SCFHS) guide was used [25]. The tools were developed through experts, the SHC team, and a scientific committee represented by all healthcare-providing entities, the Ministry of Finance, the General Authority for Statistics (GASTAT), and the Saudi Commission for Health Specialties (SCFHS).

After developing the survey, a pilot test of the questionnaire was performed before launching it nationwide. For the pilot, a random sample of all healthcare facility types were chosen across the country. It was checked for technical challenges, structure and sequence of questions, clearness of questions, any skip patterns, and the time required to complete it. At the end, a report was compiled categorizing each problem into common themes. Based on these results, the survey tools were revised and then approved by the team.

The surveys were administered by trained interviewers in all 13 administrative regions of Saudi Arabia. A supervisor accompanied each team of interviewers to ensure a smooth flow of data collection and to address any challenges. Within each administrative region, areas were classified into urban or rural, based on Municipal and Rural Affairs in Saudi Arabia (MOMRA) guidelines [26].

The survey team approached more than 2400 PHCCs from the master list. Data were collected from 2319 PHCCs; nearly 81 PHCCs were not cooperative. For various reasons, 75 facilities were revealed to be inactive; reasons included being merged with another PHCC or being temporarily closed for maintenance. The final analytical sample consisted of 2244 PHCCs. All the PHCCs were governmental, with nearly 95% of them being the Ministry of Health (MOH) facilities. PHCCs were distributed across all 13 administrative regions.

### Construction of service availability and drug indices

The aim of the analysis was to shed light on the differences across health facilities in terms of their readiness to provide services to patients. To do this, in addition to the variables readily available such as the numbers of health workers and examination rooms, two indices were constructed to get a more holistic inference.

The Service Availability Index is the sum of all services that the facility is able to provide. This is the sum of dummy variables for the availability of the services: Immunization, Family Medicine, Diagnostic and Basic Dental services, Chronic Disease Management including Diabetic Care, Maternal and Child Care, Obstetrics & Gynecology, Health Education, Pediatrics, Fracture Casting, First Degree Burn Management, Second & Third Degree Burn Management, Nutrition, Psychiatry, Ear, Nose and Throat (ENT), Laboratory, Pharmacy, and Emergency Services. The index ranges from 0 to 17 (best).

The Drug Availability Index is the sum of all drugs available within the facility. This is the sum of dummy variables for the following types of drug: analgesic antipyretics and antimigraine; anesthetics; antacids; antipeptic ulcer; antiamoebic; antibacterials; antivirals; anthelmintics; leishmaniasis treatment drugs; antimalarials; non-steroidal anti-inflammatories; antiepileptics; antidepressants and antipsychotics, diabetic medications; antithyroid and thyroid hormones; cardiovascular drugs; lipid lowering drugs; diuretics; antiasthmatics; antidiarrheals; antiemetics; laxatives; antispasmodics; antihemorroidals; hyperuricemia drugs; antihistamines; cough syrups; drugs for skin conditions; eye, ear and nose drugs, obstetrical & gynecological drugs; antiseptics and disinfectants; and vitamins, minerals, and nutritional supplements. The index ranges from 4 to 32 (best).

### Analytical approach

In doing the analysis we follow a two-pronged approach:

1. Each of the variables of interest—including the services, workers, various measures of infrastructure, and drug availability—were summarized descriptively by region and according to rural and urban areas (Tables 1, 3, 4).
2. Ordinary Least Squares (OLS) regressions were fitted for the number of health workers, the constructed Drug Availability Index, the constructed Service Availability Index, and the number of radiology machines (Table 5). Furthermore, a logistic regression was fitted for selected services (Table 2). Both OLS and logistic regressions control for various measures in order to understand what variables correlate with the availability and readiness of facilities. The variables used to control include administrative regions, government sector, coverage area, distance to the nearest hospital, whether the facility is located in a rural or urban area, etc.

**Table 1:** Infrastructure information per administrative regions

	Administrative Region													
Variables	Albaha	Aljouf	Almadinah	Alqaseem	Aseer	Eastern Region	Hail	Jazan	Makkah	Najran	Northern Borders	Riyadh	Tabouk	Grand total
**Numbers and Densities (per 10,000 population) of PHCCs**														
**Rural N(D)**	82 (3.24)	20 (2.4)	95 (2.55)	47 (1.29)	218 (2.01)	87 (2.4)	73 (2.2)	117 (1.24)	201 (2.08)	24 (2.1)	16 (4.74)	228 (3.37)	45 (3.7)	1253 (2.2)
**Urban N(D)**	26 (1.11)	31 (0.7)	59 (0.32)	114 (1.0)	75 (0.64)	163 (0.35)	27 (0.6)	44 (0.6)	132 (0.17)	45 (0.93)	23 (0.67)	210 (0.27)	42 (0.52)	991 (0.36)
**Numbers and Densities (per 10,000 population) of Examination Rooms**														
**Rural N(D)**	192 (7.6)	55 (6.6)	158 (4.2)	202 (5.6)	622 (5.7)	272 (7.7)	268 (8.3)	336 (3.6)	568 (5.9)	69 (6.1)	22 (6.5)	488 (7.2)	223 (18.4)	3475 (6.1)
**Urban N(D)**	94 (4.0)	135 (3.1)	246 (1.4)	341 (3.1)	327 (2.8)	768 (1.6)	154 (3.9)	157 (2.4)	776 (1.0)	197 (4.1)	108 (3.2)	1406 (1.8)	293 (3.6)	5002 (1.8)
**Percentage Distribution of Building Ownership**														
**Rural %**	43.9	80.0	37.9	72.3	49.1	56.3	68.5	57.3	52.7	91.7	62.5	52.2	60.0	54.2
**Urban %**	61.5	58.1	39.0	89.5	62.7	60.1	70.4	59.1	46.2	66.7	78.3	67.1	73.8	63.6
**Percentage of PHCCs Having Laboratory Room within the Facility**														
**Rural %**	42.7	45.0	15.8	14.9	28.9	32.2	26.0	33.3	33.3	16.7	43.8	7.9	6.7	40.0
**Urban %**	73.1	51.6	81.4	14.9	48.0	42.3	59.3	90.9	86.4	31.1	78.3	43.3	33.3	74.2
**PHCC Facilities Working 24 × 7**														
**Rural %**	2.4	50.0	62.1	0.0	22.9	11.5	32.9	55.6	15.9	16.7	81.3	0.9	13.3	22.1
**Urban %**	7.7	29.0	11.9	4.4	1.3	4.3	14.8	0.0	12.1	6.7	52.2	9.1	21.4	9.5
**Paper-Based Medical Recording**														
**Rural %**	82.9	78.9	98.8	40.0	77.6	97.6	91.5	90.4	90.7	91.7	100	93.4	93.2	87.5
**Urban %**	80.0	74.2	87.9	55.9	86.3	89.2	50.0	75.0	81.6	86.7	82.6	69.5	81.0	78.3

Note: *N* numbers, *D* density per 100,000 population

**Table 2:** Select Services available in facilities

		(1)	(2)	(3)	(4)	(5)	(6)	(7)	(8)	(9)	(10)	(11)
VARIABLE		Immunization	Family Medicine	Diagnostic and Basic Dental services	Chronic Disease Management	Maternal and Child Care	Obstetrics & Gynecology	Health Education	Pediatrics	First degree Burn Management	Ear, Nose, and Throat (ENT)	Emergency Services
Region	Makkah	5.133*** (1.621)	2.209*** (0.387)	5.318*** (1.008)	7.502*** (1.698)	6.545*** (1.277)	3.591*** (0.717)	5.803*** (1.112)	1.764*** (0.340)	1.896*** (0.406)	7.103*** (1.750)	18.03*** (3.884)
	Al Madinah	7.585*** (4.169)	0.216*** (0.0913)	7.760*** (2.162)	0.438** (0.143)	0.762 (0.260)	0.623 (0.271)	0.519 (0.224)	0.344** (0.168)	0.219** (0.137)	2.370*** (0.682)	0.746 (0.309)
	Eastern Region	1.375 (0.340)	0.725 (0.153)	3.872*** (0.821)	4.255*** (1.002)	2.232*** (0.454)	0.855 (0.228)	0.578** (0.144)	0.668 (0.170)	7.178*** (1.633)	7.538*** (2.429)	1.099 (0.278)
	Al Qaseem	0.487*** (0.105)	2.674*** (0.551)	0.559** (0.136)	0.802 (0.160)	0.764 (0.163)	2.625*** (0.588)	0.0836*** (0.0397)	3.012*** (0.632)	0.290*** (0.115)	0.547*** (0.112)	0.235*** (0.0975)
	Aseer	2.665*** (0.740)	2.663*** (0.496)	3.147*** (0.632)	7.298*** (1.757)	6.711*** (1.405)	4.796*** (0.996)	5.631*** (1.145)	1.743*** (0.355)	1.681** (0.390)	5.012*** (1.210)	10.98*** (2.377)
	Najran	11.31*** (8.310)	0.340*** (0.118)	5.351*** (1.620)	0.408*** (0.122)	0.0746*** (0.0453)	0.119*** (0.0922)	1.062 (0.328)	0.105*** (0.0785)		1.183 (0.370)	63.18*** (29.92)
	Tabuk	0.497** (0.141)	0.988 (0.281)	1.344 (0.411)	0.0637*** (0.0310)	0.439** (0.149)	3.016*** (0.885)	1.355 (0.414)	3.313*** (0.931)		0.266*** (0.0772)	3.355*** (0.987)
	Northern Borders	2.523 (1.489)	2.25e-07 (0.000117)	5.385*** (2.346)	0.697 (0.421)	1.134 (0.708)	0.292 (0.283)		0.233 (0.234)		2.174* (0.979)	
	Jazan		0.825	2.084**	2.301**	3.741***	2.462***	3.276***	1.916*	2.517**	1.325	2.977***
			(0.257)	(0.687)	(0.823)	(1.342)	(0.837)	(1.167)	(0.636)	(1.008)	(0.463)	(1.106)
	Al Jouf	2.846** (1.438)	0.656 (0.243)	8.535*** (3.138)	0.0335*** (0.0344)	1.347 (0.515)		0.175** (0.132)	0.0630** (0.0760)		2.906*** (1.116)	0.171* (0.176)
	Al Baha	6.076*** (3.236)	2.355*** (0.588)	5.227*** (1.368)	30.67*** (16.73)	19.83*** (7.136)	7.091*** (1.886)	10.10*** (2.858)	3.483*** (0.896)	6.132*** (1.654)		10.60*** (2.943)
	Hail	0.579** (0.152)	4.575*** (1.153)	2.068*** (0.554)	0.742 (0.188)	3.258*** (0.843)	0.868 (0.306)	0.0764*** (0.0573)	2.323*** (0.605)	0.185*** (0.113)	1.374 (0.351)	0.465* (0.196)
Urban		0.722* (0.125)	2.436*** (0.320)	3.294*** (0.445)	1.609*** (0.243)	2.377*** (0.345)	1.659*** (0.245)	1.931*** (0.300)	1.339** (0.190)	0.992 (0.167)	2.016*** (0.311)	1.074 (0.173)
MOD		1.415 (1.594)	2.875 (2.554)	0.870 (0.769)	0.0791*** (0.0740)	0.736 (0.650)	2.501 (2.237)	0.407 (0.504)	4.776* (4.064)	1.277 (1.153)	0.271 (0.312)	8.555** (7.762)
MOI		1.173 (1.341)	1.269 (1.285)	1.102 (1.186)		0.860 (0.808)	1.731 (2.073)	0.825 (0.973)	58.46*** (84.23)		0.231 (0.253)	
NGHA		0.505 (0.481)	6.857e+ 06 (3.561e+ 09)	1.702 (1.708)	4.857 (5.757)	0.375 (0.461)	30.38*** (31.62)	4.932 (6.172)	12.90** (13.38)			0.291 (0.656)
Accreditation		1.499** (0.260)	1.120 (0.146)	0.932 (0.129)	0.627*** (0.0890)	0.667*** (0.0946)	0.955 (0.145)	0.832 (0.134)	1.718*** (0.244)	0.557*** (0.107)	0.456*** (0.0642)	0.603*** (0.103)
Radial distance of coverage (km)		0.999 (0.00142)	1.001 (0.00110)	1.003*** (0.00113)	1.007*** (0.00163)	0.998* (0.00122)	0.999 (0.00128)	1.004*** (0.00131)	0.999 (0.00124)	0.999 (0.00141)	1.007*** (0.00182)	1.003** (0.00146)
Working 24 × 7		0.810 (0.190)	0.563*** (0.0994)	0.360*** (0.0638)	0.0675*** (0.0150)	0.0834*** (0.0195)	0.371*** (0.0836)	0.107*** (0.0299)	0.217*** (0.0515)	0.162*** (0.0600)	0.113*** (0.0210)	0.146*** (0.0351)
Distance to the closest hospital (in km)		1.002 (0.00247)	0.999 (0.00192)	0.998 (0.00194)	1.006** (0.00224)	1.003 (0.00204)	1.002 (0.00213)	1.000 (0.00223)	1.006*** (0.00198)	1.011*** (0.00236)	1.004* (0.00217)	1.005** (0.00218)
Constant		3.753*** (0.733)	0.349*** (0.0568)	0.178*** (0.0316)	0.705** (0.118)	0.376*** (0.0638)	0.149*** (0.0292)	0.237*** (0.0444)	0.191*** (0.0343)	0.166*** (0.0345)	1.207 (0.204)	0.181*** (0.0360)
Observations		1690	1778	1777	1773	1778	1731	1747	1778	1550	1681	1743

Note: *MOD* Ministry of Defense, *MOI* Ministry of Interior, *NGHA* National Guard Health Affairs

Standard errors in parentheses *** *p* < 0.01, ** *p* < 0.05, * *p* < 0.1

We utilize both approaches to be able to understand differences in resource allocation and how resource allocation can be improved. The first approach helps compare resource allocation across regions and across rural and urban areas. Regressions help us understand which factors play a role in such a distribution in order to be able to influence resource allocation through these factors.

### OLS model

Equations 1–4 analyze the factors that can explain the service availability and drug availability indices and the number of health workers and radiology machines, where *X*_*i*_ is a vector of facility-level controls such as administrative region, sector, geographic classification, coverage area, etc. for facilities 1 − N. *ε*_*i*_, *ζ*_*i*_, *ω*_*i*_ and *υ*_*i*_ are stochastic error terms.

1 $$ {Service\ Availability}_i={\beta}_0+{\beta}_1{X}_i+{\varepsilon}_i,\mathrm{with}\ i=1,\dots, \mathrm{N} $$

2 $$ {Drug\ Availability}_i={\alpha}_0+{\alpha}_1{X}_i+{\zeta}_i,\mathrm{with}\ i=1,\dots, \mathrm{N} $$

3 $$ {Health\ Workers}_i={\gamma}_0+{\gamma}_1{X}_i+{\omega}_i,\mathrm{with}\ i=1,\dots, \mathrm{N} $$

4 $$ {Radiology\ Machines}_i={\psi}_0+{\psi}_1{X}_i+{\upsilon}_i,\mathrm{with}\ i=1,\dots, \mathrm{N} $$

In order to ensure the OLS model estimates were robust, heteroscedasticity and multicollinearity were tested and accounted for. Heteroscedasticity was accounted for using the Breusch-Pagan/Cook-Weisberg test, which indicated heteroscedastic data; thus, robust standard errors are reported. Multicollinearity was also evaluated with the variance inflation factor (VIF). For all models fitted, the VIF was less than 2, so no model correction was needed [27, 28].

### Logistic regression

For the services available within each facility, which is indicated using an indicator variable equaling 1 if the facility offers this service to patients and 0 otherwise, a logistic regression was fitted.

A linear model was assumed where the main variables of interest (the service being offered) can be analyzed by regressing them (*y*_*i*_) on a vector of *k* facility-level explanatory variables (*X*_*k*_) and ε_i_ is an error term (Eq. 5).

5 $$ {Service}_{k,i}=\alpha +\sum \limits_k{\beta}_k{X}_{k,i}+{\varepsilon}_i,\mathrm{with}\ i=1,\dots, \mathrm{N} $$

Assuming that *y*_i_^*^ in Eq. (5) is a latent variable, the logit model is written as:

$$ \left\{\begin{array}{c}1\  if\ {y}_i^{\ast }>0\\ {}0, otherwise\end{array}\right. $$

## Results

### Infrastructure

The descriptive statistics on the infrastructure information per administrative region is presented in Table 1. On the national level, based on 2017 data and not accounting for rural and urban population levels, there are 0.74 PHCCs per 10,000 population in Saudi Arabia. Overall, there are variations in the distribution of the PHCC both across regions and within regions across rural and urban areas. The results show that, compared to urban areas, rural areas have more facilities (56%) in Saudi Arabia. Rural areas also have higher PHCC densities (2.20) than urban areas (0.36), owing to the very small rural population (only 17% of the population live in rural areas) [25]. Of the total number of PHCCs across all regions, Riyadh region has the highest number of PHCCs (438), followed by Makkah (333) and the Eastern Region (250). Urban areas have more examination rooms (5002); however, rural areas have higher densities of examination rooms (6.1 per 10,000 population).

In terms of the distribution of building ownership, urban areas have a higher percentage of ownership (as opposed to renting facility space) (64%). On the other hand, some remote areas, such as Aljouf (80%) and Najran (92%), exhibit a higher percentage of building ownership, which could be explained by lower land costs.

The highest percentage of laboratory rooms was reported in Jazan, followed by Makkah, Almadinah, Northern Borders, and Albaha. Urban areas have more laboratory rooms across all regions (74%).

Offering 24 × 7 services in PHCCs seems to be infrequent across the entire country, with rural areas having a higher percentage of facilities working 24 × 7 (22%). Finally, dependency on paper-based medical recording is common across KSA, with 87.5 and 78.3% of facilities in rural and urban areas, respectively, relying on paper-based medical recording.

### Serveries

Table 2 shows the odds ratio of the logistic regression, regressing each of the services being provided on facility-level controls. The most significant explanatory variables affecting the service being provided are region, whether the facility is operating 24 × 7, and accreditation status. Regarding the distribution of services across the regions, PHCCs in Makkah and Aseer are more likely to offer all services. However, other regions (i.e. Almadinah, Eastern Region, and Northern Borders) do not offer some essential services (e.g. obstetrics & gynecology and emergency services). Urban areas are more likely to offer general services but less likely to offer specific specializations such as burn management and emergency services. Coverage area and distance to closest hospital did not positively or negatively impact the likelihood of services being offered. Sectors were also mostly insignificant. Accreditation status and operating 24 × 7 had some of the greatest effect, with 24 × 7 operation negatively affecting the likelihood of offering all services, implying a tradeoff between 24 × 7 operation and comprehensiveness of services. However, accreditation increases the likelihood of offering pediatrics and immunization services. OLS Regression results in Table 5 also show that accreditation has a positive impact on the availability of radiology machines, emphasizing the importance of accreditation in resource availability and oppressiveness of services offered.

### Human resources

Overall, in terms of specialties (Table 3), PHCCs are staffed by very general specialties: mostly general medicine, family medicine, and obstetrics & gynecology. In terms of numbers, healthcare workers are more concentrated in urban areas in all fields, especially in nursing (10,417) and general medicine (3049). However, when considering densities, rural areas have higher numbers of workers per population across all specialties, mostly in nursing (83.9) and General Medicine (31) per 100,000 population. Psychiatrists and nutritionists though are very rare to find in PHCCs and do not exist in some regions.

**Table 3:** Numbers and densities (per 100,000 population) of health workers, by Specialty

	Administrative Regions													
Variable	Albaha	Aljouf	Almadinah	Alqaseem	Aseer	Eastern Region	Hail	Jazan	Makkah	Najran	Northern Borders	Riyadh	Tabouk	Grand total
**General Medicine Residents**														
**Rural N (D)**	103 (41)	38 (46)	121 (33)	64 (18)	262 (24)	113 (32)	88 (27)	173 (18)	380 (40)	47 (42)	31 (92)	270 (40)	76 (63)	1766 (31)
**Urban N (D)**	41 (18)	61 (14)	175 (10)	200 (18)	192 (16)	411 (9)	88 (22)	105 (16)	534 (7)	128 (27)	108 (32)	841 (11)	165 (20)	3049 (11)
**Family Medicine Residents**														
**Rural N (D)**	13 (16)	1 (1)	2 (1)	5 (5)	16 (9)	14 (2)	42 (13)	12 (2)	51 (9)	11 (2)	0 (0)	54 (9)	3 (11)	224 (7)
**Urban N (D)**	21 (6)	23 (3)	27 (1)	76 (8)	12 (3)	43 (2)	31 (6)	18 (3)	76 (1)	10 (2)	0 (1)	183 (1)	17 (2)	537 (2)
**Dental Residents**														
**Rural N (D)**	27 (10.7)	13 (15.6)	38 (10.2)	15 (4.1)	69 (6.4)	34 (9.7)	20 (6.2)	25 (2.6)	78 (8.1)	11 (9.7)	2 (5.9)	25 (3.7)	9 (7.4)	366 (6.4)
**Urban N (D)**	16 (6.8)	31 (7.1)	77 (4.2)	25 (2.3)	59 (5.0)	172 (3.7)	12 (3.0)	34 (5.2)	193 (2.5)	34 (7.0)	18 (5.3)	100 (1.3)	29 (3.6)	800 (2.9)
**Psychiatry Residents**														
**Rural N (D)**	0 (0)	0 (0)	0 (0)	0 (0)	0 (0)	0 (0)	0 (0)	0 (0)	0 (0)	0 (0)	0 (0)	0 (0)	0 (0)	0 (0)
**Urban N (D)**	1 (0.4)	2 (0.5)	0 (0)	2 (0.2)	4 (0.2)	9 (0.1)	0 (0)	0 (0)	13 (0.2)	0 (0)	0 (0)	8	0 (0)	25 (0.1)
**Nurses**														
**Rural N (D)**	309 (122.0)	164 (196.8)	325 (87.4)	64 (17.6)	781 (71.9)	410 (116.5)	312 (96.8)	549 (58.0)	881 (91.0)	130 (114.9)	31 (91.8)	654 (96.8)	162 (133.9)	4772 (83.9)
**Urban N (D)**	149 (63.7)	341 (78.0)	709 (39.0)	346 (31.7)	625 (53.1)	1936 (41.4)	270 (68.6)	360 (54.8)	1771 (22.6)	278 (57.6)	292 (85.5)	2722 (35.0)	618 (76.3)	10,417 (37.6)
**Laboratory Personnel**														
**Rural N (D)**	24 (9.5)	13 (15.6)	16 (4.3)	4 (1.1)	96 (8.8)	24 (6.8)	18 (5.6)	54 (5.7)	70 (7.2)	25 (22.1)	5 (14.8)	10 (1.5)	3 (2.5)	362 (6.4)
**Urban N (D)**	22 (9.4)	24 (5.5)	83 (4.6)	17 (1.6)	45 (3.8)	90 (1.9)	27 (6.9)	55 (8.4)	260 (3.3)	16 (3.3)	36 (10.5)	181 (2.3)	28 (3.5)	884 (3.2)
**Public Health Specialists**														
**Rural N (D)**	3 (1.2)	0 (0)	29 (7.8)	4 (1.1)	11 (1.0)	40 (11.4)	57 (17.7)	11 (1.2)	31 (3.2)	8 (7.1)	0 (0)	90 (13.3)	10 (8.3)	294 (5.2)
**Urban N (D)**	6 (2.6)	8 (1.8)	23 (1.3)	22 (2.0)	38 (3.2)	67 (1.4)	9 (2.3)	13 (2.0)	54 (0.7)	11 (2.3)	2 (0.6)	74 (1.0)	8 (1.0)	335 (1.2)
**Nutritionists**														
**Rural N**	0	0	0	0	1	0	0	1	2	0	0	0	0	4
**Urban N**	2		2	0	2	7	2	0	12	0	0	28	0	55

Note: *N* numbers, *D* density per 100,000 population

The OLS Regression results in Table 5 also show a large positive effect on total health workers, with accredited facilities on average having approximately four more workers working in the facility. Urban areas also have a much larger workforce, with facilities in urban areas having approximately 10 more health workers on average. Because this could be demand driven, medical records is used to control for the size of facility.

### Drug availability

Table 4 shows the availability of medications that are available in less than 95% of PHCCs. Information on other medications—such as antacids, antibacterials, diabetic medications, diuretics, asthma medications, antiemetics, antihistamines, cough syrups, and vitamins, minerals, and nutritional supplements—was collected in the survey, but was not analyzed because they are almost always available. Except for anesthetics, antimalarials, and antiepileptics, 85–100% of pharmacies within PHCCs had these medications available. Across all medications, pharmacies operating in rural areas are less likely to have certain drugs available. Although most of the medications were available overall in rural and urban areas, with availability ranging from 50 to 100%, the difference in the availability of the same medication between both areas is less than 12%.

**Table 4:** Availability of select medication in urban and rural areas per administrative regions

	Administrative Regions													
	Albaha	Aljouf	Almadinah	Alqaseem	Aseer	Eastern Region	Hail	Jazan	Makkah	Najran	Northern Borders	Riyadh	Tabouk	Grand total
**Anesthetics**														
**Rural%**	59.8	0.0	97.9	8.5	74.3	72.4	15.1	81.2	55.2	87.5	6.3	28.5	55.6	55.9
**Urban%**	57.7	3.2	74.6	64.9	66.7	51.5	25.9	61.4	59.1	71.1	0.0	38.6	40.5	51.5
**Medication to treat Ulcer**														
**Rural%**	98.8	95.0	99.0	97.9	81.2	92.0	83.6	81.2	86.6	87.5	87.5	74.1	97.8	85.8
**Urban%**	96.2	100.0	98.3	98.3	93.3	92.0	74.1	88.6	85.6	95.6	100.0	84.8	95.2	91.0
**Antiamoebic**														
**Rural%**	95.0	99.0	97.9	90.4	86.2	91.8	66.7	84.1	95.8	81.3	53.1	64.4	80.6	95.0
**Urban%**	96.8	91.5	93.0	73.3	75.5	66.7	86.4	68.9	84.4	100.0	68.1	42.9	77.0	96.8
**Antivirals**														
**Rural%**	85.0	90.5	63.8	70.6	59.8	91.8	34.2	50.3	75.0	93.8	39.5	91.1	62.1	85.0
**Urban%**	96.8	71.2	86.8	78.7	74.2	88.9	22.7	72.7	64.4	91.3	68.6	83.3	73.4	96.8
**Anthelmintics**														
**Rural%**	90.0	97.9	95.7	87.6	89.7	87.7	84.6	93.0	87.5	81.3	73.7	100.0	88.0	90.0
**Urban%**	100.0	100.0	97.4	88.0	88.3	81.5	100.0	90.9	93.3	100.0	80.0	95.2	90.3	100.0
**Antimalarials**														
**Rural%**	50.0	84.2	2.1	61.5	2.3	58.9	47.0	33.3	79.2	75.0	6.1	20.0	40.4	50.0
**Urban%**	54.8	28.8	57.0	26.7	27.6	48.2	86.4	19.7	66.7	47.8	18.6	28.6	35.3	54.8
**Non-steroidal Anti-inflammatory**														
**Rural%**	95.0	99.0	68.1	91.7	85.1	78.1	72.7	87.1	91.7	87.5	61.4	100.0	82.4	95.0
**Urban%**	100.0	94.9	85.1	78.7	90.2	66.7	93.2	86.4	80.0	100.0	74.8	90.5	84.8	100.0
**Antiepileptics**														
**Rural%**	45.0	2.1	6.4	24.3	18.4	60.3	29.1	18.4	62.5	43.8	5.7	71.1	23.5	45.0
**Urban%**	38.7	27.1	54.4	26.7	36.8	70.4	40.9	37.9	64.4	43.5	23.3	50.0	37.6	38.7
**Antithyroid and Thyroid Hormones**														
**Rural%**	95.0	94.7	66.0	88.1	71.3	97.3	48.7	53.7	70.8	93.8	27.6	95.6	67.6	95.0
**Urban%**	100.0	100.0	85.1	85.3	85.9	92.6	9.1	83.3	86.7	100.0	60.0	88.1	78.8	100.0
**Lipid-lowering drugs**														
**Rural%**	95.0	99.0	93.6	99.1	89.7	100.0	99.2	79.1	95.8	87.5	77.2	100.0	90.9	95.0
**Urban%**	100.0	98.3	91.2	93.3	87.7	96.3	93.2	87.9	97.8	100.0	79.1	97.6	89.6	100.0
**Antidiarrheals**														
**Rural%**	95.0	100.0	95.7	91.7	90.8	98.6	98.3	92.0	87.5	81.3	93.9	100.0	93.9	95.0
**Urban%**	100.0	94.9	97.4	92.0	86.5	100.0	86.4	81.8	100.0	100.0	90.0	97.6	91.2	100.0

Some drugs (i.e. anesthetics and antiepileptics), however, are actually more likely to be available in rural areas. This can be explained by the need for the facility to be more readily able to provide services that are otherwise usually offered in hospitals. This finding is confirmed in Table 5 through the OLS regression results of the Drug Availability Index, where distance to the closest hospital and whether the facility operates 24 × 7 are both positively correlated with the Drug Availability Index score. This implies that the further a PHC facility is and its 24 × 7 operation, the more likely it is performing services beyond basic PHC services, and hence its need for drugs, which otherwise would be available in hospitals.

**Table 5:** Health workers, equipment, and drug availability, and service indices

	(1)	(2)	(3)	(4)
VARIABLES	Drug Availability Index	Total Number of Radiology Machines	Number of Health Workers	Service Availability Index
Makkah	2.473*** (0.319)	0.133*** (0.0457)	3.154* (1.689)	3.361*** (0.250)
Almadinah	5.282*** (0.336)	0.170*** (0.0591)	0.199 (1.105)	0.387 (0.305)
Eastern Region	3.022*** (0.413)	0.0235 (0.0491)	2.667** (1.090)	1.351*** (0.275)
Alqaseem	4.158***	−0.0663	−4.268***	−0.466
	(0.380)	(0.0509)	(0.922)	(0.315)
Aseer	4.233*** (0.289)	0.0965* (0.0560)	−2.143*** (0.753)	3.020*** (0.237)
Najran	4.937*** (0.617)	0.231** (0.106)	0.0981 (2.073)	0.197 (0.307)
Tabouk	4.396*** (0.395)	−0.000191 (0.0526)	2.415* (1.280)	−0.607 (0.427)
Northern Borders	4.284*** (0.604)	0.319** (0.127)	−1.357 (1.515)	0.00702 (0.363)
Jazan	2.062*** (0.435)	0.214*** (0.0805)	−2.854** (1.139)	1.887*** (0.331)
Aljouf	4.351*** (0.356)	0.404*** (0.133)	1.870 (1.592)	−0.195 (0.346)
Albaha	5.558*** (0.328)	0.218*** (0.0777)	−2.933*** (0.885)	4.531*** (0.329)
Hail	4.586*** (0.389)	0.351*** (0.0765)	2.521** (1.005)	−0.0258 (0.279)
Urban	1.060*** (0.209)	0.243*** (0.0370)	9.813*** (0.972)	1.151*** (0.165)
MOD	−1.008 (2.534)	−0.264*** (0.0719)	8.219 (12.33)	0.132 (0.893)
MOI	2.393*** (0.763)	0.466 (0.370)	2.786 (4.075)	−1.065 (1.108)
NGHA	−0.208 (1.789)	0.463 (0.319)	9.621* (5.154)	1.854 (1.432)
Accreditation	−1.531*** (0.224)	0.157*** (0.0431)	3.716*** (1.073)	−0.504*** (0.169)
Radial Distance of coverage (km)	−0.00156 (0.00214)	0.00120*** (0.000367)	0.0138* (0.00796)	0.00320** (0.00155)
Current number of active medical records			0.000463*** (0.000100)	
Distance to the closest hospital (km)	0.00595** (0.00260)	−0.00100*** (0.000342)	−0.0299*** (0.00762)	0.00716*** (0.00215)
Working 24 × 7	1.350*** (0.218)	−0.150*** (0.0459)	− 0.584 (0.939)	−2.598*** (0.206)
Constant	22.56*** (0.297)	0.0126 (0.0361)	7.805*** (0.856)	5.113*** (0.207)
Observations	1767	1778	1662	1778
R^2^	0.300	0.106	0.307	0.340

Note: *MOD* Ministry of Defense, *MOI* Ministry of Interior, *NGHA* National Guard Health Affairs Standard errors in parentheses

*** *p* < 0.01, ** *p* < 0.05, * *p* < 0.1

## Discussion

Overall results show that KSA has a large capacity in terms of its PHCC facilities. Distribution is an issue, however, especially when it comes to health workers, because some regions seem to be extremely understaffed. Even though some administrative regions show a higher number of PHCCs per population, when it comes to the number of rooms and services, these PHCCs have fewer rooms than other regions. Nearly 56% of PHCCs are in rural areas; similarly, rural areas have higher densities of PHCCs than urban areas, which can be explained by the very small size of rural populations in Saudi Arabia (only 17% of the population lives in rural areas) [26].

Results and implications of this study are in line with previous literature. Almost every PHCC has a built-in pharmacy. However, this might change because of the reform that now aims to separate pharmacies and dispensaries from hospitals and primary care facilities and establish them on their own. Coordination is needed to ensure smooth transitions; this observation is in line with the literature emphasizing the need to reform PHCCs in Saudi Arabia [29].

Regression results showed that accreditation has a positive impact on availability of radiology machines and a large positive effect on the number of total health workers, with accredited facilities on average having approximately four more workers than non-accredited ones working in the facility. Accreditation also increases the likelihood of offering pediatrics and immunization services. This is in line with other studies, which have shown that accreditation has overall positive impacts on PHCCs in the region [30]. This is of particular policy relevance as it emphasizes the importance of accreditation in resource availability and service readiness, which emphasizes the opportunity available to improve capacity through more rigorous accreditation processes.

Dependency on paper-based medical records is high, making it difficult to integrate PHCC records across different facilities within the system. Electronic Medical Records (EMRs) are critical in healthcare settings, especially because EMRs make keeping patient history and recalling it more efficient. Furthermore, the presence of EMRs eases the communication both between physicians and patients and between physicians. The finding that EMRs are used and implemented only slightly in Saudi Arabia is also in line with other studies [31].

Even though rural areas have a higher percentage of PHCCs working 24 × 7 (22% of PHCCs in rural areas operate 24 × 7), some studies suggested that having extended PHCC working hours was not as in-demand in rural areas as it is in urban areas. The main reason for this is the higher percentage of working women in urban areas—these women are prevented by work from visiting PHCCs during regular working hours [32], and hence need additional non-business hours. However, according to health directorate interviews, further investment in extending PHCC working hours is needed, especially in rural areas distant from hospitals, where the PHCC becomes the only source of health services [23].

Primarily urban regions are more likely to offer general services but less likely to offer specific specializations such as burn management and emergency services, as shown by regression results. This can be explained by the higher dependence of larger, more urban regions on hospitals and shows the capacity of PHC facilities in less basic service provision. Facilities in Makkah are more likely to offer all services, especially emergency services, which can be explained by their need for such capacity and readiness because it is a tourism hub for pilgrimage. However, offering the service does not necessarily mean people have the ability to access it; many regions may have a service available but that services may not be easily accessible because of the lack of the resources required to receive it, according to stakeholders [23].

There is great variety across regions and within regions in the number of PHCCs and rooms readily available. Some remote regions—such as Najran, Aljouf, and Northern Borders—have very high PHCC and room densities because of their very small populations. The numbers of health workers in rural areas, despite equating to relatively high densities, are very low compared to the numbers of PHCC and examination rooms, shedding light on the issue of understaffing in rural areas. Globally, the shortage of healthcare professionals is a concern [33]. In addition to the healthcare human resources shortage, most of the existing healthcare providers in Saudi Arabia are non-Saudis [34]. This is especially an issue in rural areas where the non-Saudi turnover rate is high, and the environment is less attractive to Saudi health workers due to less attractive living conditions [23]. Furthermore, there is room for task shifting: physicians currently occupy most of the health system jobs, but some of these jobs could be performed by other cadres such as nurses. On the other hand, the supply of doctors in PHCCs is 40% lower than in hospitals, which implies that many of these physicians occupy administrative positions not related to the practice of medicine [34, 35].

Saudi Arabia’s population has been increasing rapidly in the past few years, with a rate of 2.4 in 2019 [36]. This rapid growth, along with the previous focus on tertiary and secondary care levels, the stress on investing and providing primary care decreased [23]. This explains the decreasing rate of PHCs per population and their inability to meet people’s services demand over the years [13].

Previous studies have shed light on PHC capacity in KSA in terms of accessibility and responsiveness through several household and individual satisfaction surveys. These surveys included parameters like location, waiting times, working hours, services provided and availability of health specialists in the PHCs. The results showed that most of the population are not fully satisfied with the current capacity of PHCs in Saudi Arabia and believe it should be improved [13, 36, 37]. The number of primary healthcare facilities does not meet the demand of the increased population and this problem is exacerbated in rural areas [23]. Human resources for health is also a big issue to the Kingdome’s PHC, especially in rural areas, which is a global issue. Many countries are suffering from chronic shortages in healthcare workers and the competition faced to attract foreign health workers to mitigate these shortages. Additionally, the high dependence on foreign workers becomes an even greater concern due to the high turnover rate of foreign health workers in Saudi Arabia [38]. The new health reforms within the vision 2030 the focusing is now shifted towards empowering citizens and encouraging students to enroll in healthcare professions especially females [39].

The advantage of this study is that national and collected parameters affect the capacity of PHCs from all sectors. Having such a snapshot of this level and depth is critical in guiding the future steps of refining and improving the healthcare system by knowing the current status of primary healthcare across the country. To ensure balanced distribution of PHCs and their services, there is a need for a well- defined and rounded approach working of all aspects affecting PHCs. In this approach, governance is the foundation. The role of governance is to ensure integration between the segregated different sectors that provide primary care services and to unify the decision-making process. Under sound governance, healthcare facilities’ planning in terms of location, infrastructure, and type of services offered will be more uniform and efficient. Additionally, re-assessing of the needed services is vital to ensure services are tailored to each region’s needs. Finally, workforce distribution should not only be linked to population size but should also consider other factors like population health status and needs. Drug availability might not be a priority, especially with the new health reforms of separating the outpatient pharmacies from PHC. However, medication still need to be available for urgent and on-site usage instead of referring patients to hospitals and adding pressure on them.

Results have also shown the important role, which accreditation plays in service availability and resource availability, which makes it an important policy level to be used to ensure distribution. For example, more rigorous accreditation standards and more frequent assessments can aid in ensuring facilities are operating at similar capacity. Being an umbrella for the healthcare sectors, the Saudi Health Council, the Model of Care Program and Vision Realization Office are essential for successfully implementing initiatives, which pours directly into establishing a robust health care system.

## Conclusion

With the planned reforms envisioning primary care as the center of the KSA’s health system to achieve better efficiency, it is important to determine the readiness of PHCCs on a national level to implement such reforms. The published research is very limited and looks at specific regions or sectors within the KSA, which is what this study aims to fill, by looking across regions and sectors to offer a more comprehensive overview. Using data collected from all healthcare sectors in Saudi Arabia and thirteen administrative regions, The Saudi Balanced Distribution Survey was utilized, showing the large capacity in terms of its PHCC facilities. Distribution is an issue, though especially when it comes to health workers. Each of the variables of interest—including services, workers, various measures of infrastructure, and drug availability—were summarized descriptively by region and according to area, rural or urban. OLS regressions were fitted for health workers, the constructed Drug Availability Index, the constructed Service Availability Index, and number of radiology machines, and a logistic regression was fitted for select services.

Highlighted findings include the correlation between accreditation and certain services being offered and more health workers being available, as well as the higher dependence on PHCCs in rural areas, which offer services that would otherwise be offered in hospitals in urban areas. These offer insights to areas of prioritization when considering future PHC policies, including prioritizing accreditation and increasing 24 × 7 services in rural areas. There are some limitations to the analysis owing to the challenges and limitations of the survey; for example, some health centers provide less complete data than others, especially within the private sector. This is because these facilities lack records of the requested data or were not cooperative in sharing some of the variables. This analysis offers only a background preliminary analysis on a national scale. Systematic analyses need to be performed on specific aspects of primary care, including studies on productivity and quality of care.

Urban areas are more likely to offer general services but less likely to offer specific specializations such as burn management and emergency services. This can be explained by the higher dependence of urban areas on hospitals. KSA still has a lot of potential to improve its PHCC capacity, especially to be able to meet Vision 2030 goals of primary and preventative care being the center of the healthcare system. This potential is especially evident in urban areas, which are more dependent on hospitals.

Regional variations in terms of both densities and infrastructure still need to be tackled. There is also still a lot of room for e-health improvements, given KSA’s so far relatively high dependence on paper-based medical recording. Finally, it is important to note that this study takes into account the entire health workforce, which is heavily dependent on non-Saudis, especially in rural areas.

Overall, the capacity of PHCs does not seem to meet the demands of the population, particularly when accounting for the increased demand given the current health system reforms, particularly those relating to the new Model of Care. Several initiatives under Vision 2030 are currently in place to enhance primary health centers and their services to build a sturdy healthcare system. These initiatives need to be scaled up and accelerated incorporating findings and policy levers discussed.

## Data Availability

The datasets used and/or analyzed during the current study are available from the corresponding author on reasonable request.

## Abbreviations

EMRs: Electronic Medical Records
GASTAT: General Authority for Statistics
HISs: Health Information Systems
KSA: Kingdom of Saudi Arabia
MOD: Ministry of Defense
MOH: Ministry of Health
MOI: Ministry of Interior
MOMRA: Ministry of Municipalities and Rural Affairs
NGHA: National Guard Health Affairs
OLS: Ordinary Least Squares
PHCC: primary healthcare center
SARA: Service Available and Readiness Assessment
SCFHS: Saudi Commission for Health Specialties
SDGs: Sustainable Development Goals
SHC: Saudi Health Council
VIF: variance inflation factor
